# Revisiting IDO and its value as a predictive marker for anti-PD-1 resistance

**DOI:** 10.1186/s12967-019-1784-8

**Published:** 2019-01-18

**Authors:** Peter Kim Moon, Stephanie Tran, Paras Singh Minhas

**Affiliations:** Department of Neurology and Neurological Sciences, Stanford University, 1201 Welch Road, MSLS P250, Stanford, CA 94305 USA

**Keywords:** Non-small cell lung carcinoma, PD-1, IDO, TDO2, Kynurenine pathway (KP)

## Abstract

Botticelli et al. proposed the activity of indoleamine-2,3-dioxygenase 1 (IDO) as a potential mechanism and predictive marker for primary resistance against anti-PD-1 treatment in the context of non-small cell lung cancer. However, there are a few points for the authors to address in order to strengthen their claims. First, there are many enzymes that modulate the kynurenine to tryptophan ratio, thereby calling into question their use of the ratio as a proxy for IDO activity. Second, the authors could compare IDO to other proposed markers in the literature, providing a better understanding of its predictive value.

## Main text

In their recent study, Botticelli et al. investigated the association between indoleamine-2,3-dioxygenase 1 (IDO) activity and resistance to anti-PD-1 treatment in the context of non-small cell lung cancer (NSCLC) [1]. The authors observed earlier tumor progression in individuals with higher serum kynurenine (KYN) to tryptophan (TRP), their marker for IDO activity, and suggested that IDO activity predicts resistance to anti-PD-1 treatment. To strengthen the validity of this claim however, the authors should consider investigating other modulators of KYN/TRP as well as compare IDO to other reported predictors of resistance.

Following the precedent set by previous studies [3, 13], Botticelli et al. measured serum KYN/TRP, and used this ratio and IDO activity interchangeably throughout their paper. While IDO is indeed an important modulator of this ratio, it is important to note that other enzymes significantly influence TRP and KYN levels, thereby altering the ratio and potentially confounding the authors’ interpretations [11]. For example, kynurenine pathway (KP) enzymes, kynureninase and kynurenine aminotransferases, are responsible for metabolizing KYN and forming downstream metabolites [2, 7]. More notably, tryptophan-2,3-dioxygenase (TDO2), a KP enzyme predominantly expressed in the liver, also metabolizes TRP to KYN [11]. Though IDO and its role at the intersection between TRP and KYN has been the center of attention with immunosurveillance in cancer, TDO2 has recently emerged as another prominent enzyme that can alter the KYN/TRP ratio in lung cancer [6, 9–11]. Indeed, Opitz et al. and Hsu et al. demonstrate that TDO2 is equally as effective as IDO in raising kynurenine levels within certain tumors. Therefore, given that Botticelli et al. measured general serum levels of KYN and TRP, a more comprehensive and accurate approach would have been to conduct a thorough analysis of the kynurenine pathway and examine contributions of KYN from both IDO and TDO2, among other enzymes. To conclude that one of the modulators has a more significant influence on the ratio in the context of NSCLC, the authors could measure the expression levels of each enzyme and track TRP flux in using mass-labeled intermediates [2, 4]. Such follow-up experiments would provide clarity to the questions regarding TRP metabolism and sources of anti-PD-1 resistance in NSCLC as well as clarify whether tumor-associated KYN is produced locally or systemically. Production of KYN by TDO2 and alternative sources may help explain why KYN-depletion studies with artificially engineered KP enzymes have had recent success, while specific IDO inhibitors such as epacadostat have failed phase III clinical trials [7, 14].

Furthermore, Boticelli et al.’s recent editorial offers insight into potential avenues for further investigating the predictive value of IDO [12]. In the editorial, the authors reference characteristics such as EGFR mutation state [8] and tumor mutational load [5], potential predictors of resistance that were previously investigated by other groups. Comparative analysis of these predictors would allow the authors to more rigorously assess KYN/TRP as a viable predictive marker and further evaluate the usefulness of combining several markers to more accurately predict early tumor progression as well as anti-PD-1 resistance. Therefore, to lend more credence to their assertion that IDO activity is a predictive marker for resistance, Boticelli et al. should consider other modulators of KYN/TRP and compare the predictive value of this ratio to other published markers.

## References

[1] Botticelli A, Cerbelli B, Lionetto L, Zizzari I, Salati M, Pisano A, Federica M, Simmaco M, Nuti M, Marchetti P (2018). Can IDO activity predict primary resistance to anti-PD-1 treatment in NSCLC?. J Transl Med.

[2] Cheong JE, Sun L (2018). Targeting the IDO1/TDO2–KYN–AhR pathway for cancer immunotherapy—challenges and opportunities. Trends Pharmacol Sci.

[3] Creelan BC, Antonia S, Bepler G, Garrett TJ, Simon GR, Soliman HH (2013). Indoleamine 2,3-dioxygenase activity and clinical outcome following induction chemotherapy and concurrent chemoradiation in Stage III non-small cell lung cancer. Oncoimmunology.

[4] DeBerardinis RJ, Chandel NS (2016). Fundamentals of cancer metabolism. Sci Adv.

[5] Gandara DR, Paul SM, Kowanetz M, Schleifman E, Zou W, Li Y, Rittmeyer A, Fehrenbacher L, Otto G, Malboeuf C (2018). Blood-based tumor mutational burden as a predictor of clinical benefit in non-small-cell lung cancer patients treated with atezolizumab. Nat Med.

[6] Hsu YL, Hung JY, Chiang SY, Jian SF, Wu CY, Lin YS, Tsai YM, Chou SH, Tsai MJ, Kuo PL (2016). Lung cancer-derived galectin-1 contributes to cancer associated fibroblast-mediated cancer progression and immune suppression through TDO2/kynurenine axis. Oncotarget.

[7] Labadie BW, Bao R, Luke JJ (2018). Reimagining IDO pathway inhibition in cancer immunotherapy via downstream focus on the tryptophan–kynurenine–aryl hydrocarbon axis. Clin Cancer Res.

[8] Lee CK, Man J, Lord S, Links M, Gebski V, Mok T, Yang JC-H (2017). Checkpoint inhibitors in metastatic EGFR-mutated non-small cell lung cancer: a meta-analysis. J Thorac Oncol.

[9] Opitz CA, Litzenburger UM, Sahm F, Ott M, Tritschler I, Trump S, Schumacher T, Jestaedt L, Schrenk D, Weller M (2011). An endogenous tumour-promoting ligand of the human aryl hydrocarbon receptor. Nature.

[10] Pilotte L, Larrieu P, Stroobant V, Colau D, Dolusic E, Frédérick R, De Plaen E, Uyttenhove C, Wouters J, Masereel B (2012). Reversal of tumoral immune resistance by inhibition of tryptophan 2,3-dioxygenase. Proc Natl Acad Sci USA.

[11] Platten M, Wick W, Van den Eynde BJ (2012). Tryptophan catabolism in cancer: beyond IDO and tryptophan depletion. Cancer Res.

[12] Salati M, Baldessari C, Cerbelli B, Botticelli A (2018). Nivolumab in pretreated non-small cell lung cancer: continuing the immunolution. Transl Lung Cancer Res.

[13] Suzuki Y, Suda T, Furuhashi K, Suzuki M, Fujie M, Hahimoto D, Nakamura Y, Inui N, Nakamura H, Chida K (2010). Increased serum kynurenine/tryptophan ratio correlates with disease progression in lung cancer. Lung Cancer.

[14] Triplett TA, Garrison KC, Marshall N, Donkor M, Blazeck J, Lamb C, Qerqez A, Dekker JD, Tanno Y, Lu W-C (2018). Reversal of indoleamine 2,3-dioxygenase—mediated cancer immune suppression by systemic kynurenine depletion with a therapeutic enzyme. Nat Biotechnol.

